# Zinc-Based Biodegradable Materials for Orthopaedic Internal Fixation

**DOI:** 10.3390/jfb13040164

**Published:** 2022-09-26

**Authors:** Yang Liu, Tianming Du, Aike Qiao, Yongliang Mu, Haisheng Yang

**Affiliations:** 1 Department of Biomedical Engineering, Faculty of Environment and Life, Beijing University of Technology, Beijing 100124, China; 2 School of Metallurgy, Northeastern University, Shenyang 110819, China

**Keywords:** Zinc-based biodegradable materials, orthopedic implant, biodegradability, mechanical property, biocompatibility

## Abstract

Traditional inert materials used in internal fixation have caused many complications and generally require removal with secondary surgeries. Biodegradable materials, such as magnesium (Mg)-, iron (Fe)- and zinc (Zn)-based alloys, open up a new pathway to address those issues. During the last decades, Mg-based alloys have attracted much attention by researchers. However, the issues with an over-fast degradation rate and release of hydrogen still need to be overcome. Zn alloys have comparable mechanical properties with traditional metal materials, e.g., titanium (Ti), and have a moderate degradation rate, potentially serving as a good candidate for internal fixation materials, especially at load-bearing sites of the skeleton. Emerging Zn-based alloys and composites have been developed in recent years and in vitro and in vivo studies have been performed to explore their biodegradability, mechanical property, and biocompatibility in order to move towards the ultimate goal of clinical application in fracture fixation. This article seeks to offer a review of related research progress on Zn-based biodegradable materials, which may provide a useful reference for future studies on Zn-based biodegradable materials targeting applications in orthopedic internal fixation.

## 1. Introduction

Bone fractures are becoming increasingly common with the rapid increases in aging population, traffic accidents, sports injuries and metabolic diseases [1,2,3]. Fractures have a lifetime prevalence of ~40% and an annual incidence of 3.6% [4]. The most common and burdensome fractures are lower leg fractures of the patella, tibia or fibula, or ankle [5]. Fracture healing is the process of reconstructing bone and restoring its biological and biomechanical functions [6,7]. As one of the common surgical treatments, internal fixation using screws, pins, plates, etc., provides mechanical stability for a fractured bone, allowing weight bearing, early use of the limb, and bone healing [8]. Success in fracture healing is closely related to the internal fixation implants used.

Implants used for internal fixation can be divided into several categories: wires, pins and screws, plates, and intramedullary nails or rods [9]. Staples and clamps are also used occasionally for osteotomy or fracture fixation [9]. Traditional fixation materials are generally nondegradable, including inert stainless steel (SS), titanium (Ti) and its alloys, and cobalt-chromium (Co-Cr) alloys [10]. They possess satisfactory biocompatibility, high wear resistance, and adequate mechanical strength (Table 1) [11,12,13]. However, they have notable shortcomings when being applied in fracture fixation. For example, metallic materials have much higher elastic modulus values (190–200 GPa for 316L SS, 210–240 GPa for Co-Cr alloys, and 90–110 GPa for Ti alloys) compared with bone tissues (3–30 GPa). Although a rigid fixation is required at the beginning of the healing process to provide a sufficient mechanical stability, a large discrepancy in stiffness between bone and the implant can lead to stress shielding and therefore can delay healing [14]. Even for a successful bone healing, a secondary surgery is often required to remove the implant [15].

Biodegradable materials are well suited to solve the issues above. Fixation implants made of biodegradable materials can provide a strong mechanical support of the fracture site at earlier stages of the healing process, and later on degrade naturally as the healed bone takes over the mechanical loading and their by-products can be absorbed and metabolized [16]. Degradable polymers are intended for applications in soft tissue graft fixation and meniscus repair due to their low strength [17,18,19]. Compared to polymers, Mg-based biodegradable materials have higher strength and modulus that are close to cortical bone (Table 1). Also, Mg ions released from Mg-based biodegradable implants have beneficial effects on bone regeneration [20]. Due to their appropriate mechanical property, biocompatibility and biodegradability, Mg-based metals have attracted a great deal of attention of in vitro and in vivo research during the last decades. Several Mg-based implants (bone screws, pins, plates) have been available in clinic or undergoing clinical trials [15,21]. However, the issues with an over-fast degradation rate and generation of hydrogen still need to be overcome. Additionally, current Mg alloys (ultimate tensile strength (UTS) 350 MPa) have relatively low mechanical strength and are only limited to non- or low-load-bearing applications, such as fixation of small bones and cancellous fragments, meniscus repair and soft tissue fixation [21]. Clearly, there remains a critical need for development of biodegradable materials for fixation of fractures at heavy load-bearing skeletal sites where fractures occur most frequently.

**Figure 1:**
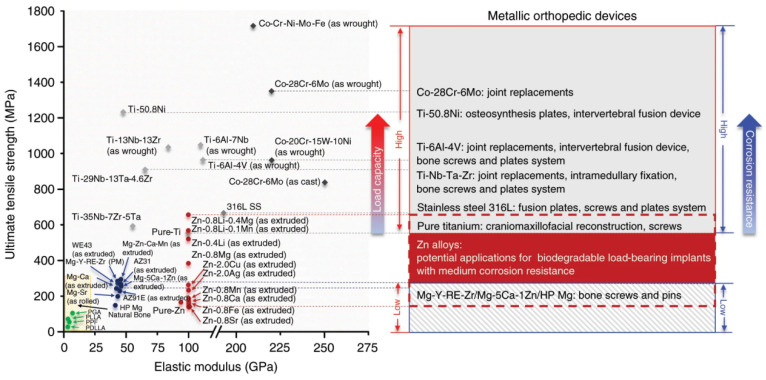
Mechanical properties of biodegradable and non-biodegradable materials for orthopaedic devices and their clinical applications [24].

The mechanical strength of zinc (Zn) alloys falls in a wide range, from the value of pure Mg to the value of commercial pure Ti and 316 stainless steel (Figure 1). For bone repair, it has been reported that the degradation rates of fixation implants should be between 0.2 and 0.5 mm y^−1^ to match bone healing [1]. Mg-based alloys have degradation rates ranging from 0.8 to 2.7 mm y^−1^ [1,15,22,23], which are above the desired degradation rates of bone implants. The degradation rates of Zn-based alloys are mainly between 0.1 and 0.3 mm y^−1^ [1,24,25]. Moderate corrosion rates and excellent mechanical properties make Zn-based biodegradable metals potential candidates for biomaterial for internal fracture fixation, particularly at heavy load-bearing sites [26,27,28]. In terms of biocompatibility, Zn is the second most abundant transition metal in humans, serving as a structural or enzymatic cofactor for approximately 10% of the proteome [29]. Consequently, perturbations in Zn homeostasis may lead to various disorders, including growth deficiencies, immune defects, neurological disorders, and cancers [30]. Studies also found that Zn ions (Zn^2+^) play an important role in promoting fracture healing [30,31]. 

**Figure 2:**
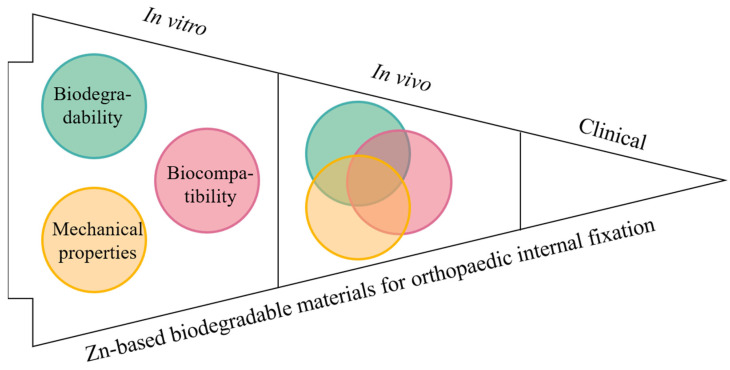
A schematic indicating the state of the art of research on Zn-based biodegradable materials for orthopaedic internal fixation.

Zn-based alloys have shown a great potential of application in orthopaedics, particularly for internal fixation of fractures at heavy load-bearing bone [24,25,32,33]. There has been a growing body of in vitro studies on the development of new Zn-based biodegradable materials and testing of their biodegradability, mechanical property and biocompatibility, with fewer in vivo animal studies and no clinical application as yet [34,35,36,37,38,39,40,41,42,43,44,45,46] (Figure 2). Although there are several review articles that have elaborated on some aspects of those properties [14,22,26,27,47], it remains unclear if current Zn-based biodegradable material are sufficient to meet clinical needs for orthopaedic internal fixation and what research gap needs to be filled next. In the following sections, we first point out the clinical requirements of implant biomaterials for orthopaedic internal fixation primarily at the heavy load-bearing skeletal sites in terms of their biodegradability, mechanical property and biocompatibility, and then summarize various typical Zn-based biodegradable materials (pure Zn, Zn-based alloys and composites) that have been developed so far and examined in vitro and in vivo for each of these properties. Lastly, unaddressed questions or future research directions are discussed with the aim of moving towards clinical applications of Zn-based biodegradable materials for orthopaedic internal fixation.

**Table 1:** Characteristics of different typical metallic biomaterials.

Classification	Materials	Biodegradability	Mechanical Properties	Biocompatibility	Applications or Potential Applications	Ref.
Non-biodegradable metallicmaterials	316L SS	Non-biodegradable	High elastic modulus, low wear and corrosion resistance, high tensile strength	High biocompatibility	Acetabular cup, bone screws, bone plates, pins, etc.	[11]
	Co–Cr alloys	Non-biodegradable	High elastic modulus, high wear and corrosion resistance	Low biocompatibility	Bone screws, bone plates, femoral stems, total hip replacements, etc.	[12]
	Ti alloys	Non-biodegradable	Poor fatigue strength, light weight	High biocompatibility	Dental implants, bone screws, bone plates, etc.	[11,13]
Biodegradable metallic materials	Mg-based alloys	Biodegradable, high degradation rate	Poor mechanical properties, elastic modulus are close to cortical bone	High biocompatibility, H_2_ evolution	Bone screws, bone plates (non-load bearing parts), etc.	[2,9]
	Fe-based alloys	Biodegradable, low degradation rate	High elastic modulus, poor mechanical properties	Low biocompatibility	Bone screws, bone plates, etc.	[9]
	Zn-based alloys	Biodegradable, moderate corrosion rate	High elastic modulus, high mechanical properties, low creep resistance	Cytotoxicity, no gas production, high biocompatibility	Bone screws, bone plates (load-bearing parts (potential applications)), etc.	[3,9,10]

## 2. Biodegradability of Zn-Based Biodegradable Materials

**Figure 3:**
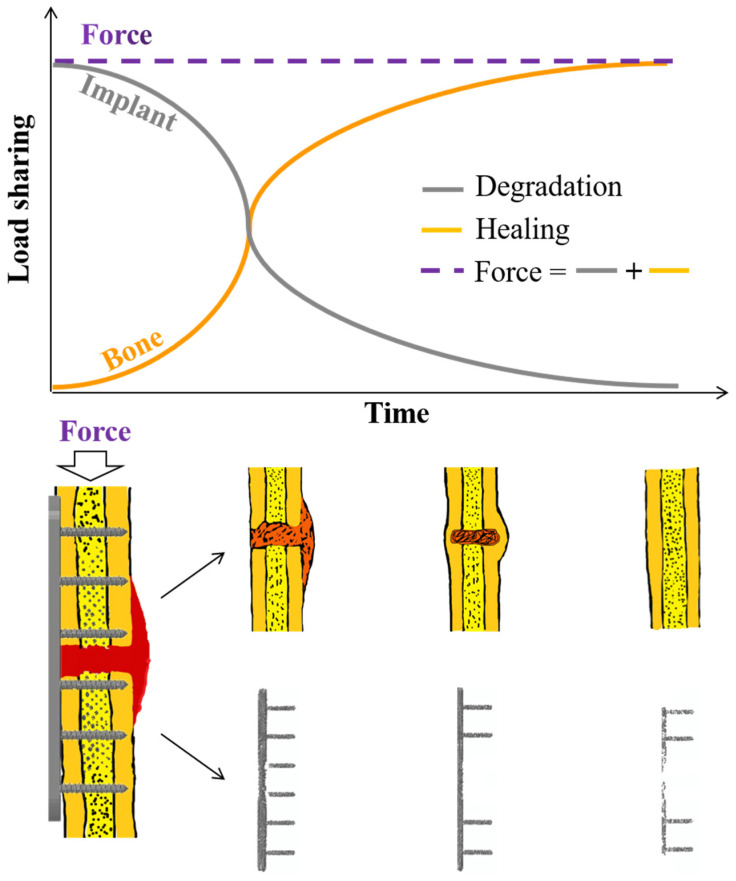
Schematics illustrating the processes of bone healing and implant degradation under a perfect matching scene.

It is well known that human bodies are full of fluid solutions and bear mechanical loading, generating corrosive and mechanical environments for biodegradable materials. It is expected that after biodegradable metals are implanted in the human body, they can gradually degrade at a suitable rate that matches the healing rate of bone tissues (Figure 3). However, different types of fractures at different skeletal sites require different fixation implants (as well as the amounts of degradable materials). Therefore, considering designs of suitable Zn-based devices with a proper degradation rate to meet clinical fixation requirements of different fractures, it is necessary to understand the degradation mechanisms, regulation of the degradation rate, and mechanical factors influencing the degradation of biodegradable metals. It is evident that pure Zn has a relatively low degradation rate. Adding alloy elements or reinforcement materials is commonly used to tune the biodegradability of Zn.

### 2.1. Biodegradability of Pure Zn

Zn is a relatively low active metal. The standard electrode potential of Zn is −0.76 V, which lies between those of Mg (−2.37 V) and Fe (−0.44 V) [14]. It is prone to corrode in various fluid environments within the human body [22]. Studies have been extensively focused on the in vitro corrosion behavior of pure Zn in different corrosion media, such as Hank’s balanced salt solution (HBSS), phosphate buffer saline (PBS), and stimulated body fluid (SBF) [16,28,48]. The medium electrode potential of Zn is associated with a moderate corrosion rate of approximately 0.1 mm y^−1^ [28]. The corrosion mechanism of Zn is regulated by the following reactions:

Zn→Zn^2+^ + 2e^−^, (1)

2H_2_O + O_2_ + 4e^−^→4OH^−^, (2)

2Zn + 2H_2_O + O_2_→2Zn(OH)_2_, (3)

Zn(OH)_2_→ZnO + H_2_O, (4)

Following the anode reaction, the Zn loses two electrons to generate the Zn^2+^. Cathode reaction is a process where the electrons of hydrogen dissolve oxygen reduction in the electrolyte to produce hydroxide (OH^−^). The simultaneous increases of Zn^2+^ and OH^−^ in the solution facilitate the precipitation of Zn(OH)_2_, but the Zn(OH)_2_ is unstable and may subsequently transform into a thermodynamically more stable ZnO [34,49]. It is evident from these series of reactions above that Zn does not release hydrogen gas during biodegradation like Mg, indicating one of the major benefits of Zn.

### 2.2. Biodegradability of Zn-Based Alloys

It has been reported that the degradation rate of bone implants should be somewhere between 0.2 and 0.5 mm y^−1^ to match bone healing [1]. Hence, pure Zn clearly does not meet the requirements of biodegradable orthopaedic implants. Adding other alloying elements is one way to alter the corrosion rate of biodegradable metals. To establish binary Zn alloy systems, some studies added alloying elements that are beneficial for bone health (e.g., Mg, Ca, Sr, Li, Mn, Fe, Cu, and Ag) into Zn-based alloys (Table 2) [24,32,40,42,44,50,51,52,53,54]. They used the same melting and extrusion process to prepare a variety of binary Zn-based alloys. Various alloying elements affect the corrosion rate of Zn-based alloys to different degrees [24]. Alloying can lead to an accelerated degradation, and Fe, Ag and Cu had the most significant roles in accelerating corrosion, followed by Li, Sr, Ca and Mg (Table 2) [24]. Studies have shown that corrosion rates of current Zn-based alloys are mainly between 0.1 and 0.3 mm y^−1^ [1]. The choice of Zn-based alloys generally depends on clinical demands, considering the fracture site, and the shape and size of fixators. In general, the degradation rate of the currently developed Zn alloy system is relatively slow. More ternary and quaternary Zn alloys may need to be developed to match the rate of fracture healing.

### 2.3. Biodegradability of the Zn-Based Composites

Other than alloying, adding reinforcement to form Zn matrix composites is another way to regulate the degradation rate of Zn metals. Composites including pure Zn as a matrix and hydroxyapatite (HA) as reinforcements were prepared by spark plasma sintering (SPS) [55]. A wide range of degradation rates (0.3–0.85 mm y^−1^) can be achieved by changing the concentration of HA. In addition, the immersion experiment of another beta-tricalcium phosphate (β-TCP)/Zn-Mg composite showed that the corrosion resistance of the composite is slightly decreased with the increase in β-TCP content [56].

**Table 2:** In vitro experiments of binary Zn-based alloys.

Composition (wt%)	Mechanical Properties			Corrosion Test		Cytocompatibility		Ref.
	σYS (MPa)	σUTS (MPa)	ε (%)	Corrosion Medium	Corrosion Rate (mm y^−1^)	Cell Type	Key Findings	
Zn-0.8Mg	203	301	13	MEM	0.071	U-2OS, L-929	Zn is less biocompatible than magnesium and the maximum safe concentrations of Zn^2+^ for the U-2OS and L929 cells are 120 μM and 80 μM.	[50]
Zn-1.0Ca	206	252	12.7	HBSS	0.09	MG63	Adding the alloying elements Ca into Zn can significantly increase the viability of MG63 and can promote the MG63 cell proliferation compared with the pure Zn and negative control groups.	[51]
Zn-1.1Sr	220	250	22	SBF	0.4	HOBs, hMSCs	The proliferation ability of the two kinds of cells did not decrease in the zinc alloy leaching solution. When the concentration of the leaching solution was low, the growth of the two kinds of cells was promoted.	[32]
Zn-0.4Li	387	520	5.0	SBF	0.019	MC3T3-E1	Zn-0.4Li alloy extract can significantly promote the proliferation of MC3T3-E1 cells.	[24]
Zn-5.0Ge	175	237	22	HBSS	0.051	MC3T3-E1	The diluted extracts at a concentration 12.5% of both the as-cast Zn-5Ge alloy and pure Zn showed grade 0 cytotoxicity; the diluted extracts at the concentrations of 50% and 25% of Zn-5Ge alloy showed a significantly higher cell viability than those of pure Zn.	[52]
Zn-6.0Ag	-	290	-	SBF	0.114	-	-	[44]
Zn-0.8Fe	127	163	28.1	SBF	0.022	MC3T3-E1	MC3T3-E1 cells had unhealthy morphology and low cell viability.	[24]
Zn-4Cu	327	393	44.6	HBSS	0.13	L-929, TAG, SAOS-2	Zn-4Cu alloy had no obvious cytotoxic effect on L929, TAG and Saos-2 cells.	[53]
Zn-0.8Mn	98.4	104.7	1.0	-	-	L-929	Zn-0.8Mn alloy showed 29% to 44% cell viability in 100% extract, indicating moderate cytotoxicity.	[40]
Zn-2Al	142	192	12	SBF	0.13	MG63	Cell viability decreased to 67.5 ± 5.3% in 100% extract cultured for one day, indicating that high concentrations of ions have a negative effect on cell growth. With the extension of culture time, the number of cells increased significantly.	[42]
Zn-0.0.5Zr	104	157	22	-	-	-	-	[54]

YS: yield Strength; UTS: ultimate tensile strength; SBF: stimulated body fluid; MEM: minimum essential medium; HBSS: Hank’s balanced salt solution; L-929: mouse fibroblasts; MG63: human osteosarcoma cells; HOBs: human osteoblasts; MSCs: human bone marrow mesenchymal stem cells; MC3T3-E1: mouse preosteoblasts; TAG: human immortalized periosteal cells; SAOS-2: human osteosarcoma cells; U-2OS: human osteosarcoma cells.

### 2.4. Biodegradability of Zn-Based Biomaterials under Mechanical Loading

The response of biodegradable materials to the combined effect of physiological loading and corrosion environment is an important issue in vivo since stress-induced degradation and cracking are common [57]. Particularly, for load-bearing fracture fixation where biodegradable implant undertakes loading, it is critical to understand how mechanical stress affects the biodegradation behavior of the implant.

The combination of mechanical loading and a specific corrosive medium environment can lead to sudden cracking and failure of degradable metals. This phenomenon is called stress corrosion cracking [57]. In vivo animal experiments and clinical studies have indicated the role of mechanical stress in the early failure of biodegradable implants [19,57,58,59]. Li et al. conducted slow-strain rate testing (SSRT) and constant-load immersion tests on a promising Zn-0.8 wt%Li alloy [60]. They investigated its stress corrosion cracking susceptibility and examined its feasibility as biodegradable metals with pure Zn serving as a control group. They observed that the Zn-0.8 wt%Li alloy exhibited a low stress corrosion cracking susceptibility. This was attributed to variations in microstructure and deformation mechanism after alloying with Li. In addition, compared to the “no stress” condition (0.124 mm y^−1^), the corrosion rate of the Zn-Li alloy only increased slightly under tensile stress of 11.1 MPa (0.129 mm y^−1^) and compressive stress of 17.7 MPa (0.125 mm y^−1^). Both pure Zn and Zn-0.8 wt%Li alloy did not fracture over a period of 28 days during the constant-load immersion test. The magnitude of the applied stress was close to the physiological loading condition and thus the authors proved the feasibility of both materials as biodegradable metals. So far, there are only a few experimental studies on the stress corrosion of Zn-based biodegradable materials. Since previous experimental studies have shown in degradable polymers or Mg-based alloys that the corrosion rate is affected by the loading mode (tension or compression) and magnitude [18,61,62,63,64], it is assumed that these effects also exist in Zn-based biodegradable materials. Identification of the quantitative relationships between various forms of applied loading and degradation behaviors of Zn-based materials is important for the design of load-bearing fixation implants. However, the corrosion behaviors of Zn-based biomaterials under different loading conditions need to be further explored.

Cyclic loading-induced fatigue fractures are very common in engineered metals, where the fatigue strength is further reduced in a corrosive environment [65]. It was reported that under the combined effects of stress and corrosive media, fatigue cracks propagate faster [57]. Corrosion fatigue is of primary concern for metallic internal fixation which commonly bear cyclic dynamic loads in vivo. The corrosion pit propagation rate is influenced by the magnitude of stress, frequency, and cycle number [66,67]. Previous studies have compared the compression-induced fatigue behavior of additively manufactured porous Zn in air and in revised simulated body fluid (r-SBF) [68]. The fatigue strength of the additively-manufactured porous Zn was high in air (i.e., 70% of its yield strength) and even higher in r-SBF (i.e., 80% of its yield strength). The high value of the relative fatigue strength in air could be attributed to the high ductility of pure Zn itself. The formation of corrosion products around the strut junctions might explain the higher fatigue strength of additive manufacturing Zn in r-SBF. The favorable fatigue behavior of additive manufacturing porous Zn further highlights its potential as a promising bone-substituting biomaterial. Another study found in their fatigue testing of Zn-0.5Mg-WC nanocomposites that the material survived after 10 million cycles of tensile loading when the maximum stress was 80% of the yield stress [69]. These results suggest that the Zn-0.5Mg-WC nanocomposite is a promising candidate for biodegradable materials. So far, there has been no report on the fatigue corrosion behavior of Zn-based alloys in vivo. Since the resistance of a material to fatigue and corrosion is an important consideration for designing implants, future relevant studies on Zn-based biomaterials may be required.

## 3. Mechanical Properties of Zn-Based Biodegradable Materials

In addition to the biodegradable properties, the mechanical properties of the biodegradable metals are also important considerations for designing orthopaedic implants for internal fixation. Yield strength (YS), ultimate tensile strength (UTS), elongation (ε) and elastic modulus (E) are common parameters which are used to indicate the mechanical properties of biomedical materials [11,37,70,71,72,73]. Extensive studies have determined those mechanical parameters of Zn-based biodegradable materials. The reported mechanical criteria for degradable metals (e.g., Mg-based) are UTS 300 MPa and ε 20% [22]. On the other hand, the current gold standard for medical metal materials, such as Ti and its alloys, has a tensile strength of over 600 MPa [13]. To certain extent, these criteria could provide guides of mechanical properties for development of Zn-based degradable materials. However, the requirement may vary with different load-bearing sites.

### 3.1. Mechanical Properties of Pure Zn

Pure Zn has extremely low yield strength (29.3 MPa) and elongation (1.2%) in its as-cast condition [74]. The Young’s modulus of pure Zn is around 94 GPa [16]. Obviously, it is difficult to meet the mechanical criteria as biodegradable metals [22]. On the other hand, owing to the low melting point of Zn, several additional uncertainties exist with regard to the mechanical properties of biodegradable Zn and Zn-based alloys. Low creep resistance, high susceptibility due to natural aging, and static recrystallization may lead to the failure of Zn-based biodegradable materials during storage at a room temperature and usage at a body temperature [26]. Studies showed that Zn-based alloys underwent appreciable creep deformation under human body temperature (37°) [75]. In addition, recrystallization of Zn-based alloys under stress can reduce their resistance to creep [42]. Thus, creep deformation is an important factor that should be considered in the studies of pure Zn.

### 3.2. Mechanical Properties of Zn-Based Alloys

Alloying is a common approach to change the mechanical properties of metals, where alloy ratio is essential for studies of Zn-based alloys. Attempts have been made to optimizing the Zn-based alloys by changing the alloy ratio, in order to obtain better mechanical performance in vitro and then move to in vivo conditions [24,32,40,42,44,50,51,52,53,54]. Zn-based alloys have Young’s modulus values ranging from 100 to 110 GPa depending on alloying conditions [16]. As summarized in Table 2, the Zn-based alloys with improved mechanical properties to various degrees are generated by adding elements of Mg [50], Ca [51], Sr [32], Li [24], Ge [52], Ag [44], Fe [24], Cu [53], Mn [40], Al [42], Zr [54]. The improvement of adding Li elements is particularly obvious, but the elongation of Zn-Li is only 5%. Following addition of the Cu element, the elongation of the Zn-based alloys reaches 44.6%. Binary Zn-based alloys have poor mechanical properties and may not be applicable in load-bearing sites of the skeleton. Table 3 summarizes the mechanical properties of ternary Zn-based alloys on the basis of binary Zn-based alloys.

Different mechanical processing methods have great influences on the mechanical properties of the same Zn-based alloys. Among the three common mechanical processing operations (hot extrusion, hot rolling, and casting), the hot extrusion can produce the greatest improvement in mechanical properties of Zn-based alloys. Compared with binary Zn-based alloys, ternary Zn-based alloys have largely improved mechanical properties. For example, the tensile strength of Zn-0.8Li-0.4Mg is 646 MPa, which is greater than those of pure titanium or 316L SS (Figure 1) [24]. In addition, reasonable mechanical integrity of Zn-0.8Li-0.4Mg was maintained in vitro, and is expected to be used for bone repair at load-bearing sites.

### 3.3. Mechanical Properties of Zn-Based Composites

Apart from the addition of alloying elements, adding reinforcement matrix as composite could also regulate the mechanical properties of Zn metals. The biocompatibility and the mechanical properties were improved by controlling the type and content of the second phase to form a composite material. In a previous study, Zn-HA composites were prepared with pure Zn as matrix and hydroxyapatite (HA) as reinforcement by spark plasma sintering [55]. In vivo tests showed that the addition of HA resulted in a better performance in osteogenesis with prolonged fixation time. In another study, Zn-Mg-β-TCP composites were prepared with Zn-Mg as matrix and β-TCP as enhancer by the mechanical stirring combined with ultrasonic assisted casting and hot extrusion technology [56]. This material had an ultimate tensile strength of 330.5 MPa and showed better biocompatibility than Zn-Mg alloys in cellular experiments. A barrier layer of ZrO_2_ nanofilm was constructed on the surface of Zn-0.1 wt%Li alloy via atomic layer deposition (ALD) [76]. Their results indicated that the addition of ZrO_2_ could effectively improve cell adhesion and vitality, and promote osseointegration, but the non-degradation of ZrO_2_ brought new challenges. Composites often have advantages over alloys due to the addition of second-phase enhancers. Compared with pure Zn, the addition of a second-phase material largely enhances its mechanical strength and biocompatibility. However, it was reported that the ductility of Zn-based composite materials is only 10% or even lower, with a greater brittleness [22], bringing difficulties to the processing of orthopaedic devices (such as bone screws and bone plates). In addition, the complex manufacturing process, high cost of composite materials, and a lack of sufficient basic theoretical supports in the field of preparation and processing still limit their developments.

**Table 3:** In vitro experiments of ternary Zn-based alloys.

Composition (wt%) and Manufacturing Process	Mechanical Properties			Corrosion Test		Cytocompatibility		Ref.
	σYS (MPa)	σUTS (MPa)	ε (%)	Corrosion Medium	Corrosion Rate (mm/y)	Cell Type	Key Findings	
Zn-1.5Mg-0.5Zr HE	350	425	12	-	-	L-929	Overall, the L-929 cells exhibit polygonal or spindle shape, and well spread and proliferated in the extracts of pure Zn and Zn alloys.	[39]
Zn-1.0Ca-1Sr Cast	86	140	1.2	SBF	-	MG63	Adding the alloying elements Mg, Ca and Sr into Zn can significantly increase the viability of MG63 and can promote the MG63 cell proliferation compared with the pure Zn and negative control groups.	[77]
Zn-1.0Ca-1Sr HE	212	260	6.7	SBF	0.11			
Zn-1.0Ca-1Sr HR	144	203	8.8	SBF	-			
Zn-0.8Li-0.4Mg HE	438	646	3.68	-	-	-	-	[24]
Zn-3Ge-0.5Mg Cast	66.9	88.3	1.4	HBSS	0.062	MG63	The extract with a concentration of 100% had obvious cytotoxicity to MG63 cells. When the concentration of the extract was diluted to 12.5% or lower, the survival rate of MG-63 cells was all above 90%.	[78]
Zn-3Ge-0.5Mg HR	253	208	9.2	HBSS	0.075			
Zn-4Ag-0.6Mn HE	-	302	35	HBSS	0.012	-	-	[79]
Zn-1Fe-1Mg Cast	146	157	2.3	SBF	0.027	-	-	[80]
Zn-0.8Mn-0.4 Cast	112	120	0.3	-	-	-	-	[68]
Zn-0.8Mn-0.4 HE	253	343	8	-	-			
Zn-0.8Mn-0.4 HR	245	323	12	-	-			

HE: Hot extrusion; HR: Hot rolling.

## 4. Biocompatibility of Zn-Based Biodegradable Materials

**Figure 4:**
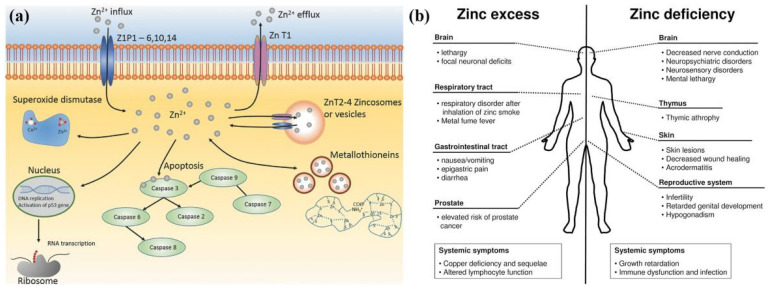
(**a**) Biological roles of Zn [81]. (**b**) Comparison of the influence of zinc excess versus deficiency [82].

Biocompatibility is the ability of a material to conform to the host response, cell response, and living systems, and it is a vital property of metallic internal fixation for bone repair [1]. The metallic fixation implants directly release ions into the human body, affecting the surrounding cells, tissues, and blood (Figure 4) and leading to either positive or negative results [47,81,82]. Additionally, biomaterial-induced infections are one of the leading causes of implant failure in orthopaedic surgery [25]. Postoperative wound infection may cause an increase in the cost of pain treatment and even sequelae such as limb malformation and dysfunction of the implants [26]. Thus, exploring the biocompatibility of Zn and its alloys is important considering their ultimate implanting in the human body.

### 4.1. Biocompatibility of Pure Zn

**Figure 5:**
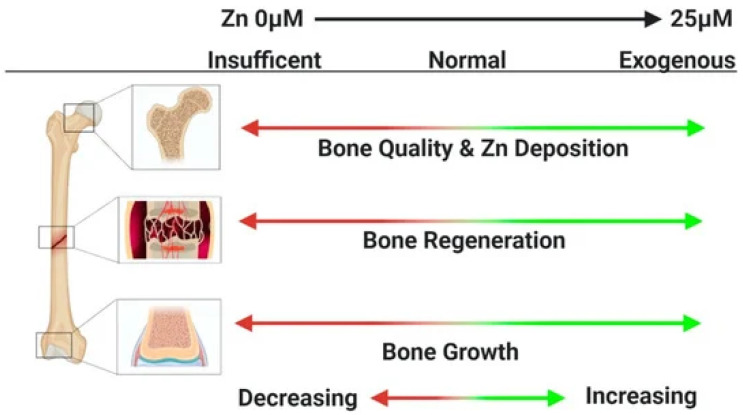
Zn^2+^ deficiency has been linked to impaired skeletal development and bone growth in humans and animals [83].

Zn plays a fundamental role in multiple biochemical functions of the human body, including cell division, cell growth, wound healing, and the breakdown of carbohydrates [83]. Dietary Zn^2+^ deficiency has been linked to impaired skeletal development and bone growth in humans and animals (Figure 5) [83]. Specifically, 85% of Zn in the human body is found in muscle and bone, 11% in the skin and liver, and the rest in other tissues [84]. Zn is located at sites of soft tissue calcification, including osteons and calcified cartilage. Zn levels in bone tissue increase as bone mineralization increases. The skeletal growth was reduced during Zn deficiency. Zn plays a key biological role in the development, differentiation and growth of various tissues in the human body [85], including nucleic acid metabolism, stimulation of new bone formation, signal transduction, protection of bone mass, regulation of apoptosis, and gene expression [14]. Zn not only inhibits related diseases such as bone loss and inflammation, but also plays an important role in cartilage matrix metabolism and cartilage II gene expression [86]. The following symptoms are associated with Zn deficiency, including impaired physical growth and development in infants and young adults, the increased risk of infection, the loss of cognitive function, the problems of memory and behavioral, and learning disability. However, excessive Zn may cause neurotoxicity problems [87]. Based on the RDI (Reference Daily Intake) values reported for mature adults, the biocompatibility of Zn (RDI: 8–20 mg/day) is not as good as that of Mg (RDI: 240–420 mg/day), but very similar to that of Fe (RDI: 8–18 mg/day) [88]. Excessive Zn can cause symptoms such as nausea, vomiting, abdominal pain, diarrhea, fatigue, and can weaken immune function and delay bone development [87]. Therefore, when Zn-based biodegradable materials are implanted into the body as bone implant materials, the toxicity of their degradation products should be considered.

### 4.2. Biocompatibility of Zn-Based Alloys

The results of cytotoxicity tests can reflect the biological safety of the material to some extent. Table 2 and Table 3 summarize the results of cytocompatibility testing of alloying elements. Specifically, according to the cytocompatibility testing, additions of Mg, Ca and Li do not produce cytotoxicity, but can promote cell proliferation. However, Cu, Al, and Fe show varying degrees of toxic effects on bone cells [34,37,40,42,43,73,74,89].

Regarding the effect of metal ions on antibacterial activity, Sukhodub et al. [90] systematically examined the antibacterial abilities of metal ions and reported that the sterilization rate of the metal ions from high to low was as follows: Ag^+^, Cu^2+^, Zn^2+^, and Mg^2+^. Among these metal ions, Zn ions have a good antibacterial ability when they reach a certain concentration and can kill various bacteria and fungi. Zinc is an essential element with intrinsic antibacterial and osteoinductive capacity [91]. Zn-based antimicrobial materials generally consist of zinc complexes and ZnO nano-particles. Complexes such as zinc pyrithione and its derivatives are well known antifungal compounds and have been broadly applied in medicines [92]. Lima et al. [93] prepared Zn-doped mesoporous hydroxyapatites (HAps) with various Zn contents by co-precipitation using a phosphoprotein as the porous template. They found that the antibacterial activity of the HAps samples depended strongly on their Zn^2+^ contents. Tong et al. [94] examined the bacterial distributions of the Zn-Cu foams pre- and post-heat treatment after co-culturing with staphylococcus aureus for 24 h, and observed good antibacterial properties of the Zn-Cu foams. Lin et al. [74] observed better antibacterial properties of Zn-1Cu-0.1Ti than pure Zn. Ren et al. [95] systematically investigated a variety of Cu-containing medical metals including stainless steels, Ti alloys, and Co-based alloys, and demonstrated good antibacterial abilities of those materials stemming from the durable and broad-spectrum antibacterial characteristics of Cu ions. Therefore, Cu-containing Zn alloys may be expected to be promising implant materials with intrinsic antibacterial ability.

### 4.3. Biocompatibility of Zn-Based Composites

HA is a well-known bioceramic with bioactivity that supports cell proliferation, bone ingrowth and osseointegration. HA has similar chemical and crystallographic structures to bone, which can form a chemical bond with osseous tissue, and act like nucleation for new bone [17]. Yang et al. [55] fabricated Zn-(1, 5, 10 wt%) HA composites using the SPS technique and investigated their in vitro degradation behaviors. Zn-HA composites showed significantly improved cell viability of osteoblastic MC3T3-E1 cells compared with pure Zn. An effective antibacterial property was observed as well. As a bioactive ceramic, β-TCP has good biocompatibility, osteoconductivity and biodegradability [96]. In a study by Pan et al. [56], the biocompatibility of Zn-1Mg-xβ-TCP (x = 0, 1, 3, 5 vol%) composites were investigated. When L-929 and MC3T3 cells were cultured in different concentrations for one day, the relative proliferation rate of the cells is above 80%, and the cytotoxicity is 0–1. Moreover, the addition of β-TCP makes the compatibility of the composite material to MC3T3 cells significantly higher than that of the Zn-Mg alloy.

## 5. In Vivo Evaluation of Zn-Based Biodegradable Materials with Animal Models

In addition to in vitro testing, in vivo animal experiments are a necessary step in assessing the performances of Zn-based biodegradable materials prior to translation into clinical applications. Different from in vitro experiments where the biodegradability, mechanical property, and biocompatibility of a material are often tested separately, animal models can be used to examine all these properties together in an in vivo condition. Although the in vivo animal experiments may not be able to fully mimic the mechanical, biological and chemical environments in the human body, they are currently the best way to evaluate the interactions between Zn-based biodegradable materials and host [15,24,25,36,97]. There are far fewer in vivo studies on Zn-based biomaterials than in vitro studies. Several representative in vivo studies on Zn-based biodegradable materials were summarized in Table 4.

**Figure 6:**
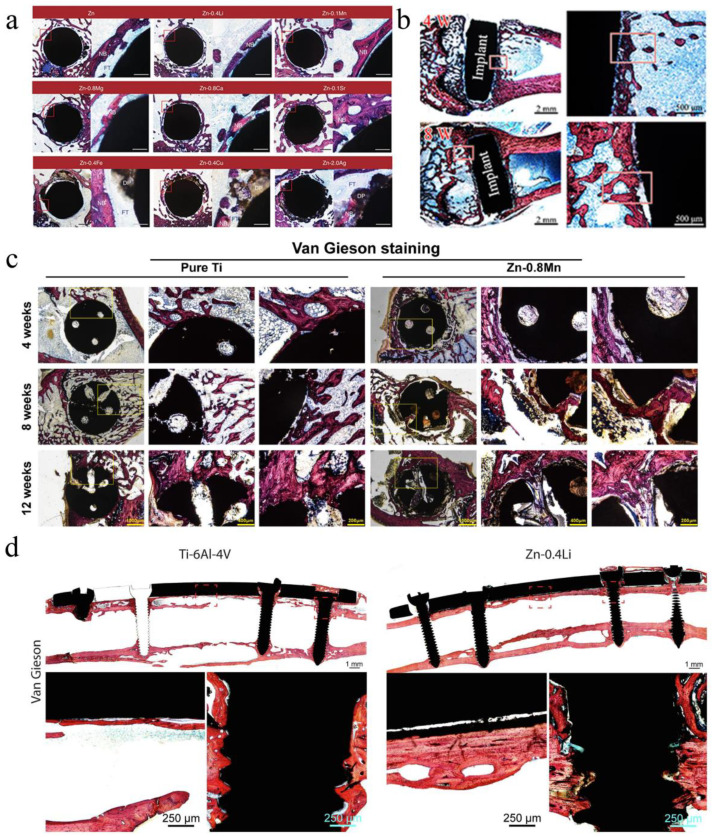
(**a**) Hard tissue sections of pure Zn, Zn-0.4Li, Zn-0.1Mn, Zn-0.8 Mg, Zn-0.8Ca, Zn-0.1Sr, Zn-0.4Fe, Zn-0.4Cu and Zn-2Ag in metaphysis. The magnified region is marked by red rectangle. NB, new bone; DP, degradation products; FT, fibrous tissue. Scale bar, 0.5 mm in low magnification, 500 μm in high magnification [24]. (**b**) Histological characterization of hard tissue sections at implant sites. Van Gieson staining of pure Zn [55]. (**c**) The Van Gieson staining results of specimens 4 weeks, 8 weeks, and 12 weeks postoperatively. Within each row, full-view images of bone defect areas (20×), medium magnification images (50×), and higher magnification images (100×) arranged from left to right [70]. (**d**) Van Gieson staining of representative histological images of femoral fracture healing at 6 months. The fracture healing and fixation screws are magnified and marked by red rectangles [25].

Yang et al. [24] implanted the pure Zn into the rat femur condyle. A serious fibrous tissue encapsulation was found for pure Zn, resulting in the lack of direct bonding between bone and implant (Figure 6a). The delayed osseointegration of pure Zn is claimed to be attributed to the local high Zn ion concentration. Consistent with the observations in vitro, the in vivo results confirmed that alloying with appropriate elements such as Mg, Ca and Sr can effectively improve the biocompatibility. Yang et al. [55] implanted the pure Zn into the rat femur condyle. A serious fibrous tissue encapsulation was found for pure Zn, resulting in the lack of direct bonding between bone and implant (Figure 6b). Meanwhile, Jia et al. [70] implanted the Zn-0.8 wt.%Mn alloy into the rat femoral condyle for repairing bone defects with pure Ti as control. Their results showed that the new bone tissue at the bone defect site in both groups gradually increased with time, but a large amount of new bone tissue was observed around the Zn-0.8Mn alloy scaffold (Figure 6c). More importantly, in a heavy load-bearing rabbit shaft fracture model, the Zn-0.4Li-based bone plates and screws showed comparable performance in bone fracture fixation compared to the Ti-6Al-4 V counterpart whereas the cortical bone in the Zn-0.4Li alloy group was much thicker (Figure 6d). The results suggest the great potential of Zn-Li based alloys for degradable biomaterials in heavy load-bearing applications [25].

It can be seen from those in vivo studies above (Table 4) that the Zn-based biodegradable materials play an important role in promoting osteogenesis. The corrosion rate of Zn-based biodegradable materials is relatively slow in vivo and can provide a long-term mechanical support in the period of fracture healing [24,25,36]. No incomplete fracture healing and structural collapse of the implant were reported during the animal experiments on load-bearing parts such as the femur [25]. However, the long-term results of the Zn-based implants remain unknown since those animal studies generally lasted for 8–24 weeks [24,25,46]. Additionally, the in vivo studies testing performances of Zn-based implants in fracture fixation are limited [24].

**Figure 7:**
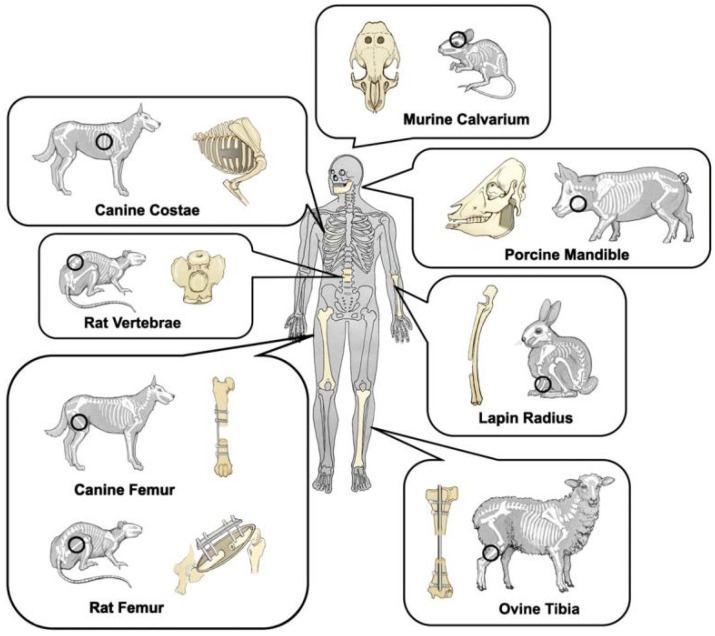
Schematic representation of common animal bone defect models [98].

Currently, only small animal models, such as mice, rats and rabbits have been used to examine primarily the biodegradability and biocompatibility of Zn^2+^ metals on bone defect sites (Table 4) [24,25,33,36,46,51,55,56,70,97]. Although mammals have many similarities, differences across small animals, large animals and humans should be recognized [98]. For example, the difference in skeletal size across various species affects the amount of Zn materials that needs to be degraded or absorbed as well as the mechanical environment, which may lead to varied results between preclinical studies and clinical applications. Therefore, with clinical translations in mind, future studies may be warranted with large animal models. In addition, as Zn-based implants are expected to be used at heavy load-bearing sites for internal fracture fixation, proper site-specific in vivo animal models should be used to test their biodegradability, mechanical properties and biocompatibility (Figure 7).

**Table 4:** Relevant animal studies of Zn-based biodegradable materials as potential orthopaedic implants.

Zn-Based Metals	Designed Implants	Control	Surgeries	Animal Species	Major Findings	Ref.
Zn-Mn	Scaffold	Pure Ti	Insertion into femoral condyle	Rats	The new bone tissues at the bone defect sites gradually increased with time in both groups, and numerous new bone tissues were observed around the Zn-0.8Mn alloy scaffold	[70]
Zn-1Mg, Zn-1Ca, Zn-1Sr	Intramedullary nails	NA	Insertion into femoral marrow medullary cavity	Mice	There was no inflammation observed around the implantation site and no mouse died after operation. The new bone thickness of Zn-1Mg, Zn-1Ca and Zn-1Sr pin groups are significantly larger than the sham control group.	[51]
Zn-HA	Pin	Pure Zn	Insertion into femoral condyle	Rats	There was new bone formation around the Zn-HA composite, and the bone mass increased over time. With prolonged implantation time, the Zn-HA composite was more effective than pure Zn in promoting new bone formation.	[55]
Zn-0.05Mg	Pin	Pure Zn	Insertion into femoral condyle	Rabbits	No inflammatory cells were found at the fracture site, and new bone tissue formation was confirmed at the bone/implant interface, proving that the Zn-0.05Mg alloy promoted the formation of new bone tissue.	[46]
Zn-(0.001% Mg 2.5%, 0.01% Fe 2.5%)	Screw and plate	PLLA, Ti-based alloys	Mandible fracture	Beagles	The new bone formation in the Zn alloy group and the titanium alloy group was significantly higher than that in the PLLA group. In addition, the new bone formation in the Zn-based alloys group was slightly higher than that in the Ti-based alloys group. The degradation of Zn implants in vivo would not increase the concentration of Zn^2+^.	[97]
Zn-X (Fe, Cu, Ag, Mg, Ca, Sr, Mn, Li)	Intramedullary nails	Pure Zn	Insertion into femoral marrow medullary cavity	Rats	Pure Zn, Zn-0.4Fe, Zn-0.4Cu and Zn-2.0Ag alloy implants showed localized degradation patterns with local accumulation of products. In contrast, the degradation of Zn-0.8Mg, Zn-0.8Ca, Zn-0.1Sr, Zn-0.4Li and Zn-0.1Mn was more uniform on the macroscopic scale.	[24]
Zn-0.8Sr	Scaffold	Pure Ti	Insertion into femoral condyle	Rats	Zn-based alloys promote bone regeneration by promoting the proliferation and differentiation of MC3T3-E1 cells, upregulating the expression of osteogenesis-related genes and proteins, and stimulating angiogenesis.	[36]
Zn-0.8Li-0.1Ca	Scaffold	Pure Ti	Insertion into radial defect	Rabbits	The Zn-0.8Li-0.1Ca alloy has a similar level of biocompatibility to pure titanium, but it promotes regeneration significantly faster than pure Ti.	[33]
Zn-0.4Li	Screw and plate	Ti-6Al-4V	Femoral shaft fracture	Rabbits	Plates and screws made of Zn-0.4Li alloy showed comparable performance to Ti-6Al-4V in fracture fixation, and the fractured bone healed completely six months after surgery.	[25]
Zn-1Mg-nvol%β-TCP (*n* = 0, 1)	Columnar samples	Zn-1Mg	Specimens in lateral thighs.	Rats	Zn-1Mg alloy and Zn-1Mg-β-TCP composites had no significant tissue inflammation and showed good biocompatibility.	[56]

## 6. Summary and Future Directions

**Figure 8:**
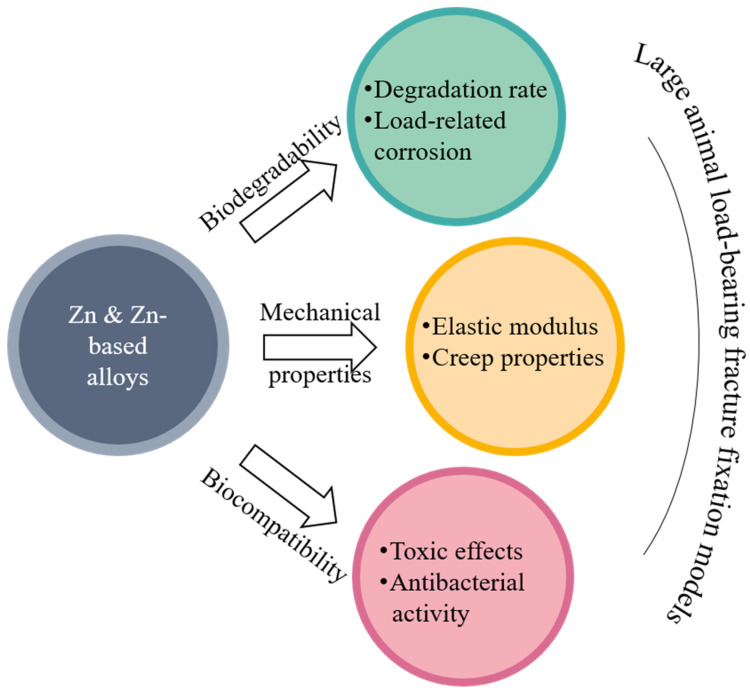
Future directions of Zn-based biodegradable materials.

A growing number of new Zn-based biodegradable materials have been developed and their biodegradability, mechanical properties, and biocompatibility were tested mostly in vitro and partially in vivo. An ideal biodegradable material for orthopaedic internal fixation should have a suitable combination of biocompatibility, biodegradability, and mechanical properties (YS, UTS, and ε). Although the mechanical properties of pure Zn are difficult to meet the requirements of orthopaedic fixation, Zn-based alloys can achieve the mechanical properties of traditional implants used in internal fixation at load-bearing sites. Zn-based materials have a moderate corrosion rate and good biocompatibility. Their degradation by-product Zn^2+^ can promote bone growth and mineralization. These properties support Zn-based biodegradable materials as an alternative for internal fixation implants at heavy load-bearing skeletal sites. However, many questions still need to be addressed before Zn-based biodegradable materials can be used for fracture fixation in clinics (Figure 8).

In terms of biodegradability, (1) the current degradation rate of Zn-based biodegradable materials remains relatively slow, and it needs to be further tuned according to the target skeletal site to match its healing rate; (2) due to existence of static and dynamic loads on the skeleton, the stress corrosion and fatigue corrosion of the materials need to be better understood. In terms of the mechanical properties, (1) the elastic modulus (94–110 GPa) of current Zn-based biodegradable materials is higher than that of bone and should be reduced to avoid stress shielding when being used in internal fixation; (2) the creep effect of Zn-based alloys on the failure of the internal fixation implant at a physiological temperature of the human body should be explored further. In terms of biocompatibility, (1) since high content of Zn^2+^ has toxic effects on cells, attempts should be made to regulate the degradation rate of internal fixation to ensure that the concentration of degradation products does not exceed the safe concentration range of the implant site; (2) antibacterial properties may be explored further. Additionally, in vivo experiments should move from small animal models to large animal models for heavy load-bearing fracture fixation.

## Data Availability

Not applicable.
